# Nitric Oxide-Dependent Activation of CaMKII Increases Diastolic Sarcoplasmic Reticulum Calcium Release in Cardiac Myocytes in Response to Adrenergic Stimulation

**DOI:** 10.1371/journal.pone.0087495

**Published:** 2014-02-03

**Authors:** Jerry Curran, Lifei Tang, Steve R. Roof, Sathya Velmurugan, Ashley Millard, Stephen Shonts, Honglan Wang, Demetrio Santiago, Usama Ahmad, Matthew Perryman, Donald M. Bers, Peter J. Mohler, Mark T. Ziolo, Thomas R. Shannon

**Affiliations:** 1 Davis Heart and Lung Research Institute, Department of Physiology and Cell Biology, The Ohio State University, Columbus, Ohio, United States of America; 2 Department of Molecular Biophysics and Physiology, Rush University, Chicago, Illinois, United States of America; 3 Department of Pharmacology, University of California Davis, Davis, California, United States of America; University of Canberra, Australia

## Abstract

Spontaneous calcium waves in cardiac myocytes are caused by diastolic sarcoplasmic reticulum release (SR Ca^2+^ leak) through ryanodine receptors. Beta-adrenergic (β-AR) tone is known to increase this leak through the activation of Ca-calmodulin-dependent protein kinase (CaMKII) and the subsequent phosphorylation of the ryanodine receptor. When β-AR drive is chronic, as observed in heart failure, this CaMKII-dependent effect is exaggerated and becomes potentially arrhythmogenic. Recent evidence has indicated that CaMKII activation can be regulated by cellular oxidizing agents, such as reactive oxygen species. Here, we investigate how the cellular second messenger, nitric oxide, mediates CaMKII activity downstream of the adrenergic signaling cascade and promotes the generation of arrhythmogenic spontaneous Ca^2+^ waves in intact cardiomyocytes. Both SCaWs and SR Ca^2+^ leak were measured in intact rabbit and mouse ventricular myocytes loaded with the Ca-dependent fluorescent dye, fluo-4. CaMKII activity in vitro and immunoblotting for phosphorylated residues on CaMKII, nitric oxide synthase, and Akt were measured to confirm activity of these enzymes as part of the adrenergic cascade. We demonstrate that stimulation of the β-AR pathway by isoproterenol increased the CaMKII-dependent SR Ca^2+^ leak. This increased leak was prevented by inhibition of nitric oxide synthase 1 but not nitric oxide synthase 3. In ventricular myocytes isolated from wild-type mice, isoproterenol stimulation also increased the CaMKII-dependent leak. Critically, in myocytes isolated from nitric oxide synthase 1 knock-out mice this effect is ablated. We show that isoproterenol stimulation leads to an increase in nitric oxide production, and nitric oxide alone is sufficient to activate CaMKII and increase SR Ca^2+^ leak. Mechanistically, our data links Akt to nitric oxide synthase 1 activation downstream of β-AR stimulation. Collectively, this evidence supports the hypothesis that CaMKII is regulated by nitric oxide as part of the adrenergic cascade leading to arrhythmogenesis.

## Introduction

In the heart, increases in the inotropic, chronotropic, and lusitropic states are primarily brought about by the stimulation of β-adrenergic receptors (β-ARs) [1]. Upon their stimulation, signaling cascades are initiated within the myocyte that alter the way Ca^2+^ is handled and stored by the various proteins of the excitation-contraction coupling (ECC) machinery [2]. These alterations lead to an increased sarcoplasmic reticulum (SR) Ca^2+^ concentration ([Ca]_SRT_), ultimately governing the amount of Ca^2+^ made available to bind to the myofilaments and thus the strength of contraction [3].

A new paradigm involving the regulation of ECC by reactive oxygen species (ROS) and reactive nitrogen species (RNS), such as nitric oxide (NO) and peroxynitrite (ONOO^−^), has emerged. Ranging from acute to long-term regulation, the ROS/RNS axis has been shown to play an important role in controlling Ca^2+^ handling during the fight or flight reaction and the chronic pathological condition of heart failure (HF) in both humans and animal models of heart disease [4]. The extent to which these effects are related to arrhythmogenesis as a cause of or as a response to heart disease is unknown.

Activation of β-AR leads to large increases in the generation of arrhythmogenic spontaneous Ca^2+^ waves (SCaWs), especially in cells from HF animal models [5]. This increase is dependent upon calmodulin-dependent protein kinase II (CaMKII) activity. However, the activation pathway of CaMKII in response to β-AR signaling is not well understood [6]. Classically, CaMKII is thought to rely upon increases in [Ca] to initiate and maintain enzyme activity. However, recent evidence has emerged supporting novel activation mechanisms of CaMKII that are independent of increases in Ca^2+^ [7]–[12]. These mechanisms are of particular importance in HF where total cellular Ca^2+^ is low and contractility is blunted. The lower [Ca^2+^] would be expected to attenuate CaMKII activity. However, just the opposite is commonly observed; CaMKII activity in HF is high.

Here we further investigate how CaMKII activity may be maintained independent of Ca^2+^ by measuring CaMKII-dependent leak and resultant SCaW formation. We find that 1) Inhibition of nitric oxide synthase (NOS) attenuates SCaW formation as a result of β-AR stimulation in isolated rabbit myocytes; 2) the increased SCaWs are associated with an increase in RyR-dependent diastolic SR Ca^2+^ release (SR Ca^2+^ leak) and this leak is dependent upon Akt-mediated NOS1 activity in cells from rabbit and NOS1 knockout (NOS1^−/−^) mice; and 3) NO directly affects CaMKII to sustain its activity leading to the increase in SR Ca^2+^ leak. Collectively, these data indicate that NO is a signaling molecule in the β-AR cascade that activates CaMKII leading to arrhythmogenic SCaW formation.

## Materials and Methods

### Ethics Statement

Experiments were conducted in strict adherence to the guidelines for the care and use of experimental animals at Rush University Medical Center and The Ohio State University were approved by the Rush Institutional Animal Care and Use Committee (Animal Welfare Assurance, A-3120-01) and The OSU Institutional Animal Care and Use Committee (Animal Welfare Assurance, A-3261-01) conformed to the *Guide for the Care and Use of Laboratory Animals* published by NIH (publication No. 85-23, revised 1985). All animals were euthanized under deep anesthesia via rapid thoracotomy and excision of the heart. Rabbits were anesthetized using pentobarbital (I.V. into the marginal ear vein), and mice were anesthetized with Avertin (I.P.). All efforts were made to minimize any potential suffering or pain experienced by the animals.

Ventricular myocytes were isolated from New Zealand white rabbit (Myrtle Rabbitry Thompson Station, TN)and mice. WT (C57BL/6) and NOS1^−/−^ mice were acquired from Jackson Labs (Bar Harbor, MA). Data were collected with PClamp (Axon Instruments, Foster City, CA). Mathematical data manipulation was performed using Microsoft Excel (Microsoft Corporation, USA) and GraphPad Prism (GraphPad Software, San Diego, CA). All experiments were conducted at room temperature (25°C).

Chemicals and reagents were purchased from Sigma Aldrich unless indicated. Normal tyrode (NT) solution was made up as follows (all concentrations in mM): 2 Ca (1 for mouse), 140 NaCl, 4 KCl, 1 MgCl, 10 glucose, 5 HEPES, pH 7.4 with NaOH. 0 Na/0 Ca NT with caffeine was made up as NT with LiCl substituted for NaCl, with 10 EGTA, no Ca added, 10 caffeine, pH 7.4 with LiOH.

The CaMKII inhibitor KN-93, cell-permeable CaMKII inhibitor Autocamtide-2 Related Inhibitory Peptide II (AIP), NOS1 and NOS3 specific subtype inhibitors S-Methyl-L-thiocitrulline (SMLT) and L-N^5^-(1-Iminoethyl) ornithine (L-NIO), and S-Nitroso-N-acetyl-DL-penicillamine, (SNAP) were obtained from Calbiochem. Angiotensin II Type IA (ATII) was purchased from EMD Biosciences.

### Ca Measurement

All experiments were performed at room temperature using our protocol shown in Figure S1 in File S1 and as previously described [7], [13]. Before each leak protocol myocytes were stimulated electrically at 0.5 Hz for rabbit and 1.0 Hz for mice for at least 20 pulses to assure that steady state calcium handling was achieved. The diastolic whole cell fluorescence (F_0_) between beats was collected. The diastolic [Ca]_i_ ([Ca]_d_) under each relevant condition was determined in separate experiments using calibrated fura-2 fluorescence (data not shown). This [Ca]_d_ did not statistically vary between treatments, and was generally found to be approximately 120 nM. The fluo-4 fluorescence (F) during the subsequent protocol was calibrated by using a pseudoratio where K_d(Ca)_ of fluo-4 was 1.1 µM.

### SR Ca Leak Measurement

The protocol used to measure SR Ca leak in both rabbit and mouse was as previously described [7]. For a more complete discussion see supplementary materials. Briefly, [Ca]_i_ was measured using a calibrated fluo-4 (Invitrogen) signal in isolated myocytes in the presence and absence of SR Ca leak. Tetracaine was used to rapidly and reversibly block the RyR thus disrupting the SERCA pump-leak balance. The tetracaine-dependent shift of Ca from the cytosol to the SR (decrease in [Ca]_i_ and increase in SR Ca content) is proportional to SR Ca leak.

[Ca]_i_ was measured using fluo-4 fluorescence in isolated myocytes in the presence and absence of SR Ca leak flux (J_leak_). Cells were subjected to a protocol to load the SR in a graded manner: 1) by emptying the SR with 10 mM caffeine followed either by 30 sec of rest, 30 sec of rest followed by on single stimulation, or field stimulation at 0.25 Hz up to 1.0 Hz. Field stimulations at the given rates were performed at least 20 times to bring the cellular Ca content to steady-state.

After one of the above loading protocols the bath solution was rapidly switched to 0 Na, 0 Ca NT, 1 mM tetracaine. Without Na and Ca in the bath, NCX, the primary Ca efflux mechanism at rest, was blocked so that Ca was entrapped in the resting cell [14]. The RyR (and therefore leak) is blocked by tetracaine and the measured resting fluorescence decreases as Ca is taken up into the SR (Figure S1 in File S1) [7]. Fluo-4 fluorescence was corrected for a 4% quench by tetracaine whenever it was present. Fluorescence was monitored for 30 s followed by another rapid solution switch to 0Na, 0Ca NT with no tetracaine added. With the SR Ca leak restored, diastolic [Ca]_i_ rises back to its resting value. Finally, 10 mM caffeine in 0 Na, 0 Ca NT was added to cause SR Ca release. The [Ca]_SRT_ was calculated as the difference between the basal and peak total cytosolic [Ca] ([Ca]_T_) in the presence of caffeine. The difference in [Ca]_SRT_ in the presence and absence of tetracaine (the same as the difference in resting [Ca]_T_) is due to the leak dependent shift of Ca from the cytosol to the SR (i.e. the difference in basal [Ca] with and without tetracaine) and the leak rate is proportional to this shift.

### Spontaneous Ca Wave Measurement

SCaWs were assessed as previously described [5]. Fluo-4 AM (10 µM) loaded myocytes were electrically field stimulated for at least 5 minutes before data acquisition. Grading [Ca]_SRT_ was achieved by stimulating at frequencies from 0.25 Hz to 1.0 Hz in 2 Ca NT solution. After 20 beats a rapid switch to 0 Na/0 Ca NT solution+10 mM caffeine was applied for 2 seconds to empty the SR of Ca. The difference between basal and peak total cytosolic [Ca^2+^] in the presence of caffeine is therefore total SR [Ca^2+^].

After assessing [Ca]_SRT_ the myocyte was loaded under the same conditions. After loading, field stimulation was terminated and [Ca]_i_ was continuously monitored for 90 seconds. Spontaneous calcium release was determined by visual inspection, and confirmed if the peak signal was greater than two standard deviations above the average signal for the preceding 50 ms.

### In Vivo Measurement of NO Production

Myocytes were loaded with cell permeable NO-dependent fluorescent dye DAF-2 AM (10 µM) for 25 minutes, and allowed to de-esterify for an additional 25 minutes. Cells were paced at 1.0 Hz +/− isoproterenol (a β-AR agonist, ISO). Fluorescence was observed on an Olympus Fluoview confocal microscope in *x-y* mode with the pinhole set at 400 µm. Fluorescence data was acquired for 5 seconds at 1 minute intervals over the duration of 20 minutes. The dye was sensitive to photobleaching and the signal was corrected. Unstimulated myocytes without ISO present were subjected to the same experimental conditions to assess photobleach. A line was fit to this data, and the slope of this line was added back to all experimental groups to correct for bleach. SNAP was used as a positive control for DAF-2.

### In Vitro Measurement of CaMKII Activity

Purified CaMKII was incubated with 200 µM Ca and CaM for 10 min. to pre-activate the molecule. H_2_O_2_ (1 µM) or 500 µM SNAP was added and allowed to incubate for 30 min. EGTA (10 mM) was then added and allowed to incubate for 10 min. Radiolabeled ATP (^32^P) was added along with 5 µL of purified β_2a_ L-type Ca channel subunit on nickel beads. Incorporation of ^32^P into β_2a_ was allowed to proceed for 10 minutes. Phosphorylated β_2a_ is the reporter of this assay.

### S-NO Immunoblots

CaMKII was immunoprecipitated using the Classic Immunoprecipitation Kit (Pierce/Thermo Scientific). Briefly, cell lysates were pelleted with a microcentrifuge for 10 minutes and the pelleted debris was discarded. Lysates were then added to a spin column with agarose resin and incubated for 1 hour at 4°C. After incubation, CaMKII antibody was added to the flow through and incubated overnight at 4°C. The incubate was applied to a spin column with proteinA/G agarose and incubated 1 hour at 4°C. CaMKII was eluted with elution buffer and Western blotted with 1∶1000 anti-S-NO antibody.

### Statistical Analysis

Data are reported as mean ± SEM. Student *t* test was applied when appropriate. P<0.05 was considered statistically significant. To compare DAF-2 dependent fluorescence a non-parametric Spearman correlation test was conducted. The Spearman r-values are reported as an index of correlation of NO production with time.

## Results

### Inhibition of NOS Attenuates Arrhythmogenic Spontaneous Ca^2+^ Waves

We previously demonstrated that the CaMKII-dependent increased SR Ca^2+^ leak contributes to increased incidence of arrhythmogenic spontaneous SR Ca^2+^ waves (SCaW) in both healthy myocytes and those isolated from failing hearts [5], [7]. NO-mediated signaling has been demonstrated to modulate the cellular response to ISO [4]. We therefore hypothesized that NO or one of its downstream effectors or congeners (i.e. PKG or ONOO^−^) might influence CaMKII activity.

**Figure 1:**
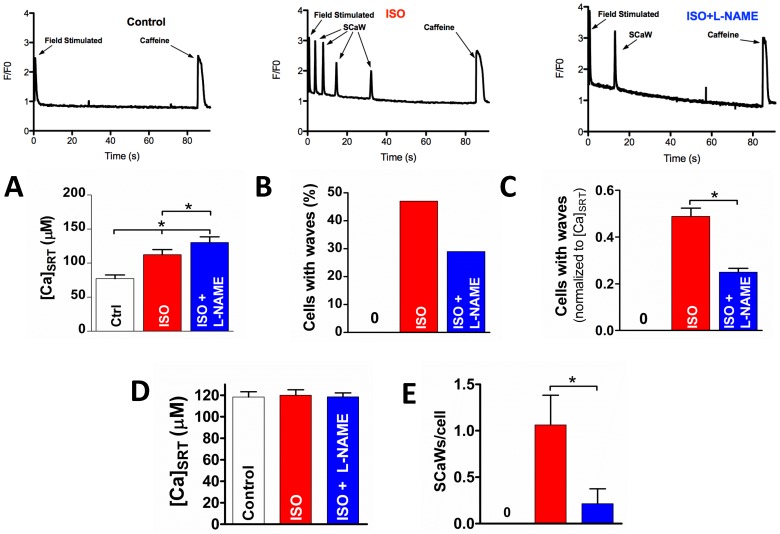
Inhibition of NOS attenuates SCaW formation in ISO treated myocytes. A) Average [Ca]_SRT_ (n = 34–40) for each treatment (raw data at the top). B) Percentage of myocytes showing at least one SCaW. C) Data in B, normalized to myocyte [Ca]_SRT_. D&E) [Ca]_SRT_ matched data (D) and the average number of SCaWs exhibited (E, n = 13–15). t-test, *p<0.05).

To test this we applied the general NOS inhibitor *N*_ω_-Nitro-L-arginine methyl ester hydrochloride (L-NAME, 100 µM) to isolated rabbit ventricular myocytes while in the presence of ISO. Figure 1A shows the average [Ca]_SRT_ from all cells examined with the percentage of those myocytes showing a SCaW activity in Figure 1B. Untreated myocytes did not show any SCaWs, but 47% were active after treatment with ISO. This activity was suppressed when treated with ISO plus L-NAME (29%). It is known that [Ca]_SRT_ regulates Ca^2+^ release [15], [16]. To control for this effect we normalized SCaW formation to [Ca]_SRT_ (Fig. 1C). After normalizing to [Ca]_SRT_, the ISO-treated cells were significantly more active (0.49±0.04) than those treated with ISO plus L-NAME (0.25±0.02). When myocytes were selected such that their [Ca]_SRT_ did not vary (Figure 1D), ISO-treated myocytes had a significantly higher number of SCaWs per cell (Figure 1E) compared to ISO plus L-NAME at the same [Ca]_SRT_. This data set implicates NOS activity in the formation of SCaW in intact ventricular myocytes stimulated by ISO.

### Adrenergic Increase in SR Ca^2+^ Leak Is Nitric Oxide Synthase Dependent

Having established the relationship between NOS activity and the generation of arrhythmogenic SCaWs, we hypothesized that the SCaWs were the result of NO-dependent increases in SR Ca^2+^ leak [5], [7]. We therefore measured SR Ca^2+^ leak as the shift of Ca^2+^ from the cytosol to the SR in response to RyR inhibition with tetracaine.

**Figure 2:**
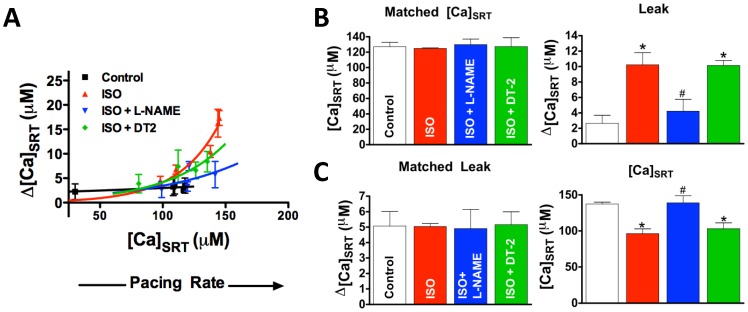
ISO-dependent leak is attenuated by NOS inhibitor, L-NAME. A) The leak-dependent shift of Ca^2+^ from the cytosol to the SR. Each point represents a loading protocol (from low to high [Ca]_SRT_; resting, 1 field stimulation, 0.25 Hz, 0.5 Hz and 1 Hz stimulation, respectively). B) The SR Ca^2+^ leak (right) in [Ca]_SRT_ matched data (left, n = 10–14). C) The [Ca]_SRT_ (right) needed to induce the same SR Ca^2+^ leak (left) in leak matched data (left, n = 11–17). *Statistically different from control, #different from ISO (t-test, p<0.05).

Figure 2A shows that treatment by 250 nM ISO alone left-shifts the leak/load relationship away from control such that more SR Ca^2+^ leak is observed at a given [Ca]_SRT_ consistent with previous data [7]. On the other hand, those myocytes stimulated by ISO with L-NAME showed a leak/load relationship shifted back towards control. Again, to control for effects of [Ca]_SRT_ on Ca^2+^ release, we matched data such that [Ca]_SRT_ was the same for both groups (127 µM, Figure 2B). Myocytes stimulated with ISO had significantly higher leak compared to control and this increase was prevented by L-NAME (10.2±1.5, 2.6±1.02, 4.2±1.5 µM Δ[Ca]_SRT_, respectively). Similarly, when selecting for myocytes such that SR Ca^2+^ leak was the same for all groups (5.1 µM, Figure 2C), the [Ca]_SRT_ needed to induce that leak was significantly lower in myocytes stimulated by ISO versus control and, again, this change was ablated in the presence of L-NAME.

**Figure 3:**
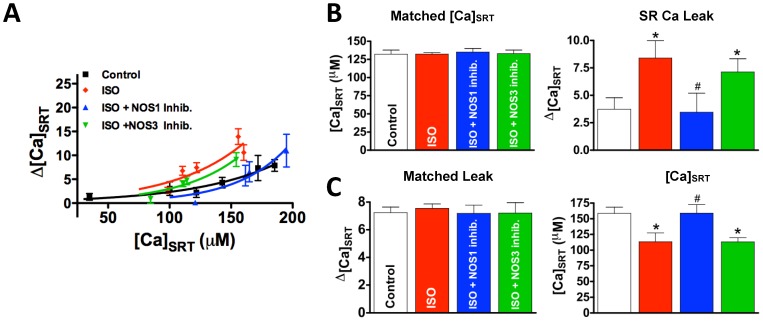
Inhibition of NOS1 but not NOS3 reverses the ISO-dependent increase in SR Ca^2+^ leak. A) Leak/load relationship. B) Matched data such that the average [Ca]_SRT_ was the same for all treatments (left) and resultant leaks (right, n = 13–17). C) Data matched such that the average SR Ca^2+^ leak was the same for all treatments (left) and the [Ca]_SRT_ needed to induce that leak (right, n = 11–19). *different from control, # different from ISO (t-test, p<0.05).

Two regulated NOS subtypes are constitutively expressed in healthy ventricular myocytes, NOS1 and NOS3 [17]. We specifically inhibited each in the presence of ISO (Figure 3). Inhibition of NOS1 by the NOS1-specific inhibitor, SMLT (3 µM), while in the presence of ISO resulted in a right-shift in the leak/load relationship away from ISO alone and towards control. Inhibition of NOS3 by L-NIO (5 µM) had no effect. Statistically, myocytes stimulated with ISO and ISO plus L-NIO had significantly higher leaks (8.3±1.6; 6.8±1.2 µM, respectively) compared with ISO plus SMLT or control (3.5±1.7; 3.7±1.0 µM, respectively) at the same [Ca]_SRT_ (Figure 3B). Similarly, cells stimulated with ISO or ISO plus L-NIO required a significantly lower [Ca]_SRT_ (113±14; 113±6.6 µM respectively) compared with ISO plus SMLT or control (159±14; 159±10 µM, respectively) to induce the same SR Ca^2+^ leak (Figure 3C, see also Supplement, Figure S2 and Table S2 in File S1).

**Figure 4:**
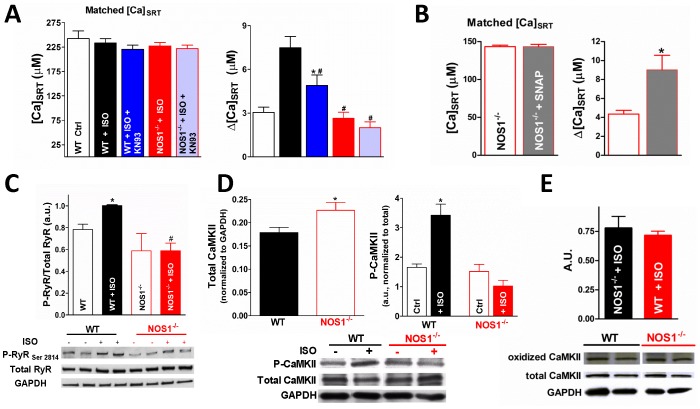
NOS1^−/−^ mice show attenuated CaMKII-dependent leak. A) Matched data such that [Ca]_SRT_ was the same for all treatments (left) an resultant SR Ca leaks (right, n = 10–22). B) Matched data such that [Ca]_SRT_ was the same in NOS1^−/−^ and NOS1^−/−^+SNAP (left) and the resultant SR Ca leaks (right), demonstrates that SNAP restores the leak/load relationship in NOS1^−/−^ myocytes. C) Summary data (top, n = 4 hearts each) and representative immunoblot (bottom) of phosphorylated RyR at CaMKII-specific residue, Ser2814, and total RyR expression in WT and NOS1^−/−^ heart lysates. D) Western blots showing total CaMKII normalized to GAPDH (left) and CaMKII phosphorylated at T286 (right, n = 5) in WT and NOS1^−/−^ hearts. Representative blot showing at bottom. E) Summary data (top) and representative immunoblot (bottom) of oxidize CaMKII after ISO stimulation in WT and NOS1^−/−^ heart lysates. *Statistically different from control, # different from WT+ISO (t-test, p<0.05).

To further validate the NOS1 dependency of leak, we measured the ISO-dependent leak in ventricular myocytes isolated from NOS1^−/−^ mice. To establish that the same CaMKII-dependent increase in SR Ca leak is present in mice, we first demonstrate that ventricular myocytes isolated from WT mice have an increased SR Ca leak in the presence of ISO and that this increase is reversed by the CaMKII inhibitor, KN93 (3.0±0.4, 7.5±0.8, 4.9±0.7 µM for control, ISO, ISO+KN93, respectively, Figure 4A). Critically, ISO treatment in myocytes isolated from NOS1^−/−^ mice was unable to increase SR Ca^2+^ leak above control levels (2.6±0.4 µM), and inhibition of CaMKII had no further effect on leak (2.1±0.4 µM). To establish that SR Ca^2+^ leak is able to be increased in the NOS1^−/−^, SR Ca^2+^ leak was measured in the presence of SNAP (an NO donor). We demonstrate that in the presence of SNAP that SR Ca^2+^ leak is increased in NOS1^−/−^ myocytes (Figure 4B). This data agrees with the previously published study of Wang et al. that extensively investigated the effect of exogenous NO on Ca handling in the NOS1^−/−^ model [18]. In line with published data, using WT myocytes we observe an increase in the degree of RyR phosphorylation at the CaMKII-dependent site, S2814, after stimulation with ISO. Critically, this increase in CaMKII-dependent phosphorylation is not present in NOS1^−/−^ mice (Figure 4C). These data demonstrate that NOS1-dependent CaMKII activity mediates SR Ca leak.

To further investigate NOS1-dependent CaMKII activation, T286 autophosphoryaltion in the NOS1^−/−^ myocytes was measured by immunoblotting (Figure 4D). ISO increased CaMKII phosphorylation in WT myocytes, and this effect was absent in NOS1^−/−^ myocytes. Total CaMKII was increased in NOS1^−/−^ myocytes compared to control (4D,left). We believe this is a compensatory mechanism to possibly attenuate the effect of decreased CaMKII activity present in NOS1^−/−^ myocytes (4C). Furthermore, we observed no differences in oxidized CaMKII between WT and NOS1^−/−^ hearts stimulated by ISO (Figure 4E). These data further support the hypothesis that ISO-dependent increases in SR Ca^2+^ leak are CaMKII-dependent and implicate NOS1/NO signaling as a necessary component of CaMKII activation.

### Stimulating Myocytes with ISO Increases NO Production

**Figure 5:**
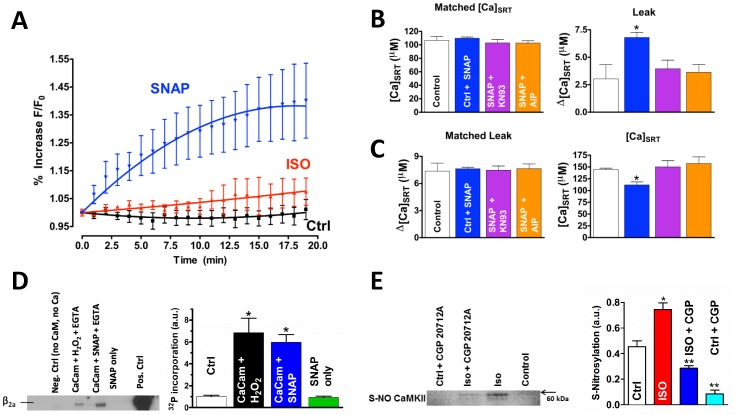
NO increases CaMKII-dependent SR Ca^2+^ leak. A) NO-dependent DAF-2 fluorescence (n = 6). Spearman correlation = 1.0 for SNAP, 0.9 for ISO, and −0.05 for control. B) SNAP-dependent SR Ca^2+^ leak. The SR Ca^2+^ leak (right) in [Ca]_SRT_ matched data (left, n = 9–13). C) Data was matched such that leak was the same (left) with the [Ca]_SRT_ needed to induced that leak shown on the right (n = 12–17). D) Purified CaMKII pre-activated with 200 µM Ca^2+^ and CaM. H_2_O_2_ (Lane 2) or 500 µM SNAP (Lane 3) was added followed by EGTA. ATP^32^ was added along with purified β2a L-type Ca^2+^ channel subunit on nickel beads. Incorporation of P^32^ was measured as an indicator of Ca-independent sustained kinase activity. Lane 1 is CaMKII without Ca^2+^, CaM, or ATP; Lane 4 is CaMKII without Ca^2+^, CaM, or ATP plus the addition of SNAP (500 µM) alone. Lane 5 is P^32^ incorporation in the continued presence of Ca^2+^ and CaM. E) Cardiac myocytes were field stimulated at 0.5 Hz under the indicated conditions. CaMKII was then immunoprecipitated from cellular homogenates which were then blotted with antibody to S-NO. *different from ISO, **different from both ISO and control (t-test, p<0.05).

To demonstrate that NO production is increased with β-AR stimulation, we tracked cellular NO (±ISO) by using the NO-dependent fluorescent dye DAF-2 in isolated rabbit myocytes. Both the NO donor SNAP (positive control) and ISO increase NO compared with control (Figure 5A, Spearman *r* = 1.0, 0.9 and −0.05, respectively). The data is in line with previous findings [19], suggesting this increased production is responsible for the observed NO-dependent effect on leak.

### NO Is Sufficient to Increase SR Ca^2+^ Leak

We stimulated rabbit myocytes with the NO donor, SNAP (100 µM), and assessed SR Ca^2+^ leak. Myocytes stimulated with SNAP had a significantly higher leak at the same load compared with SNAP plus KN93, SNAP plus the CaMKII inhibitor AIP, or control (Figure 5B; 6.8±0.5, 3.9±0.8; 3.6±0.7, 3.0±1.3 µM, respectively). The [Ca]_SRT_ needed to induce the same leak was significantly lower with the SNAP treatment versus SNAP plus KN93, SNAP plus AIP, or control (Figure 5C).

The data in Figure 5A–C demonstrate that in the absence of β-AR stimulation, NO alone is sufficient to increase SR Ca^2+^ leak and that this leak requires CaMKII activity. Though some minor SNAP-dependent effect such as direct nitrosylation of the RyR could not be completely ruled out [18], the data indicate that much of the NO effect takes place upstream of CaMKII, resulting in its activation and a subsequent increase in SR Ca^2+^ leak.

### Adrenergic Activation Leads to Reactive Nitrogen Species-dependent Sustained CaMKII Activity

Physiologically, NO often acts on target proteins by direct nitrosylation [17]. It has been shown that RyR function can be changed by S-nitrosylation via NO^+^-, N_2_O_3_^−^ or ONOO^−^-dependent action [20]. It has long been known that PKG activity is NO-dependent [17]. However, PKG inhibition with DT-2 did not alter the leak versus load relationship (see figure 2) leading us to conclude that the ISO effect upon SR Ca^2+^ is PKG-independent.

Work by Erickson, et al [8] demonstrated that CaMKII activity can be sustained by oxidation. This prompted us to investigate the possibility that NO can replicate this effect. To test this, purified CaMKII was incubated with Ca^2+^ and CaM to pre-activate the molecule. This was followed by oxidation by H_2_O_2_ or 500 µM SNAP. EGTA (10 mM) was then added to stop Ca-CaM mediated activity. Finally, ATP^32^ was added along with purified L-type Ca^2+^ channel β2a subunit on nickel beads. Incorporation of P^32^ into β2a (phosphorylation) was therefore a measure of the sustained, Ca-CaM independent activity. Ca-CaM independent kinase activity (Figure 5D) was sustained in the presence of H_2_O_2_ (as in Erickson, et al; Lane 2) and in the presence of SNAP (Lane 3) indicating that, like oxidation, NO can sustain CaMKII activity in the absence of Ca^2+^, likely by direct nitrosylation.

To investigate if CaMKII is modified by S-nitrosylation upon β-AR stimulation we isolated cardiac myocytes and field stimulated them at 0.5 Hz in the presence or absence of ISO. Total CaMKII was immunoprecipitated from cellular homogenates and was probed with an antibody against S-NO. Cellular homogenates from myocytes stimulated with ISO showed an increase in nitrosylation (Figure 5E). This increase was reversed in the presence of the β_1_ receptor blocker, CGP-120712A. Together, the data in Figure 5 indicate that NO alone is sufficient to maintain CaMKII activity and increase SR Ca leak, and upon β-AR stimulation, CaMKII is S-nitrosylated. These data support the hypothesis that NO-dependent activation of CaMKII is downstream of β-AR stimulation and increases SR Ca leak.

### The Effect of ISO upon SR Ca^2+^ Leak is Akt-dependent

**Figure 6:**
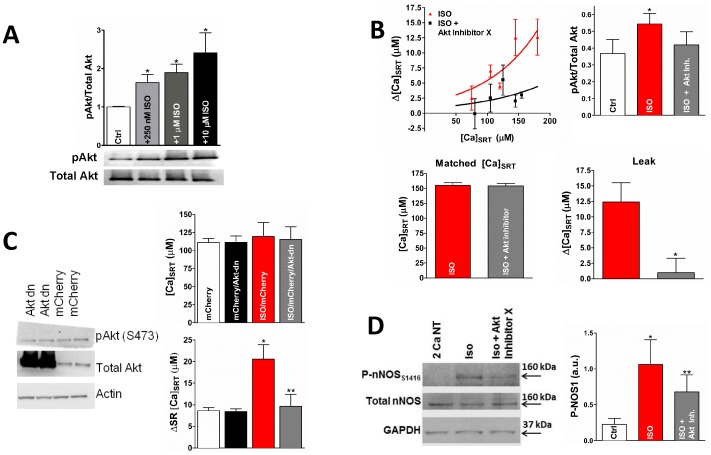
Akt Activates NOS. A) Western blot indicating that ISO increases Akt phosphorylation at S473 in a dose-dependent manner in isolated rabbit cells. B) ISO-dependent increase in p-Akt is blunted by Akt-Inhibitor X (top, right, * different from control, ** different from ISO, paired t-test, p<0.05). Cells were treated with ISO or ISO+Akt Inhibitor X. Akt Inhibitor X decreased the SR Ca leak and the activation of Akt (top left and bottom). C) Down-regulated Akt expression (plus the constitutively expressed Akt) increased the total Akt over the constitutive expression alone (top). Akt-dn decreased ISO-dependent SR Ca leak when data was selected to give the same average [Ca]_SRT_ (bottom). D) NOS1 phosphorylation at Akt phosphorylation site S1416, representative immunoblot (left) and summary data (left). (* different from control, ** different from ISO, paired t-test, p<0.05).

Finally, we set out to establish a mechanistic link between β-AR stimulation and NOS activation. Akt is a known regulator of NOS activity in numerous cell types [21]–[23]. Therefore, we tested whether Akt was involved in the NO-dependent activation of CaMKII. Akt activity (measured as S473 phosphorylation) showed a dose-dependent increase in response to ISO in rabbit myocytes (Figure 6A) which was decreased by the addition of the Akt Inhibitor X (Figure 6B).

Akt inhibitor X also prevented the ISO-dependent increase in SR Ca^2+^ leak (Figure 6B). However, because Akt-inhibitor X also severely decreased contraction in control cells, further experimentation to rule out non-specific effects was needed. Therefore, we cultured adult rabbit cardiac myocytes transfected with a dominant negative form of Akt (Akt-dn). Figure 6C (left) shows the increased total Akt in the transfected myocytes vs. endogenous expression alone in non-transfected myocytes. As we anticipated, ISO increased the SR Ca^2+^ leak in mCherry Red transfected control myocytes at the same [Ca]_SRT_. Transfection of Akt-dn along with mCherry brought the SR Ca^2+^ leak back towards control levels (Figure 6C, right). Finally, Figure 6D shows that phosphorylation of NOS1 at S1416, which is believed to be involved in the activation of NOS, is increased with ISO and this increase is reversed by Akt Inhibitor X. Taken together, the data indicate that Akt is a necessary mediator in the β-adrenergic pathway leading to increased SR Ca leak via NOS1 activation.

## Discussion

In this study we show evidence of a novel NO-dependent activation scheme for CaMKII leading to the generation of arrhythmogenic SCaWs ISO-dependent increases in RyR-dependent diastolic SR Ca^2+^ leak was observed in rabbit and mouse ventricular myocytes. This increase is dependent upon NOS1 activity but not NOS3. Further, we find that NO is sufficient to induce increased leak in the absence of ISO and that this NO-dependent effect requires CaMKII activity, indicating that CaMKII is a downstream target of NO signaling. Finally, the data indicate that Akt is activated downstream of β- AR stimulation, leading to the activation of NOS1 and the subsequent increase in both CaMKII activity (likely through nitrosylation) and CaMKII-dependent SR Ca leak. We conclude that NOS1 is a potentially important therapeutic target for the treatment of arrhythmogenic heart disease.

### NO Acting as a Regulated Signal in the β-AR Cascade

Our data lead us to conclude that the ISO-dependent increase in SR Ca^2+^ leak is mediated by a new and unique adrenergic second messenger pathway involving NO. As a result of NO production caused by β-AR stimulation, CaMKII becomes activated and mediates the increase SR Ca leak. Recent work has indicated that CaMKII can be activated by the exchange proteins activated by cAMP (EPAC) [9], [10], [24]. This protein is activated downstream of β-AR stimulation, and was a target of investigation in this study. However, we observed no effect of EPAC on the CaMKII-dependent SR Ca^2+^ leak (Figure S4 in File S1). Neither direct stimulation of EPAC by 8-CPT nor direct activation of adenylyl cyclase by 1 µM forskolin (and therefore cAMP production) induced any increase in SR Ca leak [7]. Furthermore, we found no EPAC-related differences in spark frequency or characteristics (Figure S5 and Table S1 in File S1). We conclude that the EPAC pathway is independent of the NO-dependent mechanism described by this study.

We show directly that simply treating with ISO leads to increases in NO production (Figure 5). In these experiments the response of DAF-2 during ISO stimulation is significantly lower than that invoked by SNAP. We would propose that ISO stimulation leads to an activation of NOS1 in a highly compartmentalized NO signaling domain. It is known that NOS1, CaMKII, and RyR2 are spatially coupled [25]. This localization could facilitate efficient NO-dependent signaling leading to increased CaMKII-dependent phosphorylation as indicated by our data (Figure 4C). We observed an increase in CaMKII-dependent phosphorylation of approximately 25%. This increase, though seemingly modest, results in a significant shift in the SR Ca leak. This data is in line with several previous studies that demonstrate similarly moderate increases in RyR phosphorylation can have dramatic effects on Ca handling. [26]–[29]. This likely reflects the non-linearity of Ca release [13]. The Ca release process is exquisitely tuned to respond to small shifts in [Ca]_SRT_ and changes to the Ca^2+^ sensitivity of the various Ca^2+^ proteins such as SERCA or RyR2. Our data support this in that apparently moderate shifts in RyR2 phosphorylation (i.e. Ca-sensitivity of the RyR2) results in non-linear shifts in SR Ca leak.

Recently, Gutierrez et al. reported an NO-dependent increase in CaMKII activity leading to increased SR Ca^2+^ leak and spontaneous waves using exogenous NO, remarkably similar to the results of this study [30]. However, our study significantly extends these findings by providing data directly investigating the underlying biochemical pathway mediating this activation in the intact physiological environment. We demonstrate that activation of Akt downstream of the β-AR is required for the NOS1-dependent increase in CaMKII activity. Our data provide the first evidence of a mechanistic link between β-AR stimulation and NOS1 activity. The work by Gutierrez et al. indicates that NO-dependent activation of CaMKII is likely mediated through at least one of three cysteine residues within CaMKII. Taken together, the combined results of these two studies provide compelling evidence for the complete biochemical pathway linking β-AR stimulation to NOS1 activation resulting in increased CaMKII activity.

The finding that Akt is likely mediating the observed NO-dependent effect on CaMKII activation downstream of β-AR stimulation was unexpected. This pathway will be important to address in future studies, especially upstream of Akt. We previously reported that the ISO-dependent increase in leak was conferred primarily though the (G_s_-dependent) β_1_-AR subtype [7]. The β_2_ receptor subtype and G_i_, which are also activated by ISO, are not involved in the response. Very little evidence has been demonstrated showing a link between G_s_ and NOS activation [19]. However, Mangmool, et al. (2010) [9] proposed that β-arrestin could be used as a scaffold to activate CaMKII locally at the β_1_-AR. Similar to our findings, these investigators found no CaMKII activation when β-arrestin was associated with either the angiotensin receptor (Figure S4 in File S1) or the β_2_ receptor. A similar mechanism may also be in effect here. Akt- and CaMKII-dependent signaling are well-established signaling pathways involved the electrical and structural remodeling of the myocardium associated with hypertrophy and heart failure. An interesting future direction may be to investigate how the new signaling paradigm described here may be involved in the evolution of heart failure.

### Regulation of CaMKII by Nitric Oxide

A common finding in human and animal models of HF and hypertrophy is the increased activity of CaMKII [31]–[33]. In the failing heart cellular [Ca]_T_ is lower versus non-failing hearts, leading to impaired contractility. This seems paradoxical, as one may expect lower [Ca]_T_ to lead to decreased CaMKII activity. However, Erickson and colleagues have proposed a plausible mechanism for the maintenance of CaMKII activity by ROS [8]. Our studies were unable to demonstrate a role for ROS generated by NADPH oxidase in myocytes acutely stimulated with ISO (Figure S3 in File S1). We would speculate that the ROS-dependent activity of CaMKII may only manifest itself under conditions of chronic β-AR stimulation, such as HF, where ROS production is increased and the uncoupling of NOS from NO to ROS production may exacerbate this condition [34].

Here we found that NO sustained CaMKII activity independent of Ca^2+^ (Figure 5D), likely by nitrosylation of residues within the regulatory domain, thus allowing for increased kinase activity [8]. Though the activation of CaMKII by SNAP makes nitrosylation more likely, an effect due to oxidation by other RNS cannot be completely ruled out In fact, we have previously shown that NOS1 in part signals via ONOO^−^ which can result S-nitrosylation and/or oxidation. [4]. Regardless, the extent to which this mechanism is involved in mediating other CaMKII-dependent effects (e.g., apoptosis, fibrosis, hypertrophy) upon the cell warrants future studies.

### Relevance to Cardiac Disease

The two most important downstream effectors of β-AR signaling are PKA and CaMKII. The data presented here implies that NO is acting downstream of β-AR stimulation to modulate RyR activity through CaMKII. This novel finding adds a new facet to the growing complexity of CaMKII regulation in the heart. Importantly, this mechanism provides insight into how CaMKII activity might be maintained in the absence of a sustained Ca^2+^ signal.

Phosphorylation of these cellular substrates by both PKA and CaMKII results in larger and faster [Ca]_i_ transients [35]. Our data suggest that the NOS-CaMKII pathway described here may contribute significantly to the inotropic effect of β-AR stimulation with increases in PKA activity normally being the dominant effector leading to most of β-AR related increase in [Ca]_i_ [4], [7].

On the other hand, the β-AR-dependent increase in diastolic SR Ca^2+^ leak and SCaWs is predominantly CaMKII-dependent. This increased leak is also potentially arrhythmogenic and adrenergic stimulation dramatically increases the frequency of SCaWs in cardiac myocytes in heart failure independent of [Ca]_SRT_ when compared to both control and heart failure without stimulation [5], [7]. The current study directly implicates NO in mediating this increase in arrhythmogenic activity and provides strong evidence for the underlying molecular mechanism. This data indicates NO production as a potential target for HF therapy.

To help prevent arrhythmia formation, many HF patients are treated with β-AR blockers, but this results in a decrease in the inotropic state of the tissue, preservation of which may be beneficial to the patient. Our data strongly suggest that targeted cardiac NOS1 inhibition (or other blockers unique to the described pathway) may have a selective anti-arrhythmic effect, decreasing SR Ca^2+^ leak and SCaWs while allowing the majority of the inotropic effects of the adrenergic system to remain. Such an action might provide a potent therapeutic approach to arrhythmic cardiac disease. Contrary to our findings, Cutler et al. recently reported NOS1 inhibition to be pro-arrhythmic [36]. They were able to demonstrate that loss of NOS1 activity leads to a simultaneous decrease in S-nitrosylation and an increase in oxidation of the RyR. Unlike the current study, this study was conducted in the absence of β-AR stimulation, and any dysregulation of Ca handling is more likely the result of changes in the ROS/RNS axis [37].

Independent studies have emerged that each add to the developing complexity of RyR regulation. An excellent study by Zhang et al. proposed a PKA-dependent mechanism [38]. However, this study examined the effects of chronic ISO exposure (several weeks) on CaMKII activation, whereas our study focuses on the acute effects of ISO. Moreover, Zhang et al. utilized a mouse model constitutively expressing the PKA inhibitor, PKI. This likely led to blunted Ca^2+^ handling and decreased [Ca]_i_ within the myocyte, thereby masking the potential for CaMKII-dependent effects. A recent study by Bovo et al. proposed a ROS-dependent mechanism of CaMKII activity in line with study by Erickson et al. [8], [26] This study found that SR Ca leak depended upon ISO-dependent production of ROS which increased SR Ca leak. Interestingly, this study also showed that ISO increased CaMKII-dependent phosphorylation of the RyR, an affect ablated by the presence of ROS scavengers. Critically, an experiment testing the potential link between ROS and CaMKII activation was not reported. This leaves open the distinct possibility that the ROS-dependent effect on SR Ca leak reported in this study might be mediated by the downstream activation of CaMKII, similar to our results. No study to date explicitly excludes the possibility that the proposed NO- and ROS-dependent mechanisms work in conjunction with one another to mediate SR Ca leak. Further experimental work is necessary to fully elucidate how these mechanisms interact (if at all) and the relative importance of each separate pathway.

In summary, the data presented here demonstrate that NO is acting downstream of β-AR stimulation to maintain CaMKII activity independent of Ca^2+^ leading to increased SR Ca leak and the formation of arrhythmogenic spontaneous Ca waves. To our knowledge, this is the first report of NO produced by NOS1 as a regulated second messenger in the β-AR signaling cascade and as an activator of CaMKII activity in ventricular myocytes. This finding adds a new facet to the growing complexity of CaMKII regulation in the heart and provides insight into how CaMKII activity might be maintained in the absence of a sustained Ca signal during HF.

## Supporting Information

File S1 **File includes Figures S1–S5 and Tables S1–S2.**
(DOC)

Figure S1 Schematic of leak protocol. Cartoon demonstrates how the fluo-4 dependent signal tracks changes in [Ca]_i_. The SR Ca leak is proportional to the fall in [Ca]_i_ and the resultant rise in [Ca]_SRT_ in the presence of the RyR blocker, tetracaine. The steady-state shift of Ca^2+^ from the cytosol to the SR in tetracaine is proportional to the SR Ca leak. [Ca] was 2 mM in rabbit and 1 mM in mouse.
(TIF)

Figure S2 Balance of fluxes analysis. a) All analysis was conducted in populations of myocytes in which [Ca]_SRT_ was matched such that it did not vary (173 µM, n = 6–13). b) Gain of EC coupling increases in presence of ISO regardless of treatment. c) Theoretical curves of velocity of SERCA-mediated uptake versus [Ca]_i_ generated from average determined V_max_ and K_m_ for individual myocytes (See Table 1S). Treating with NOS inhibitors yielded a trend downward from the velocity observed in ISO alone. d) Theoretical curves of velocity of NCX-mediated uptake versus [Ca]_i_ generated from average determined V_max_ and K_m_ for individual myocytes (See Table 1S). e) Average of experimentally determined velocities of SERCA-mediated Ca uptake at 250 nM [Ca]_i_. f) Average of experimentally determined velocities of NCX-mediated Ca uptake at 250 nM [Ca]_i_. (*statistically different from control, # from ISO.)
(TIF)

Figure S3 NADPH-Oxidase inhibitor is unable to shift leak vs. load relationship. A) Leak/load relationship for all treatments. B) Data were matched such that [Ca]_SRT_ did not vary (left) between treatments, resultant leaks are show (right, n = 11–12). C) Data were matched such that leak did vary (left), [Ca]_SRT_ needed to induce that leak are shown (right, n = 11–14). *Statistically different from control.
(TIF)

Figure S4 Neither EPAC activation nor Angiotensin II has an influence the leak vs. load relationship. A) Leak/load relationship for all treatments. Curves fit with a single exponential. In all data sets [Ca]_SRT_ increased as a function of pacing rate. B) Data were matched such that [Ca]_SRT_ did not vary (left) between treatments, resultant leaks are shown (right, n = 10–14). C) Data were matched such that leak did not vary (left), [Ca]_SRT_ needed to induced that leak are shown (right, n = 15–19). *Statistically different from control.
(TIF)

Figure S5 Spark measurements in rabbit ventricular myocytes in the presence and absence of EPAC activator, 8-CPT. All data were paired for any given cell, and data were acquired without a change in microscope settings. A) Representative linescan images from two different sparking cells. B) Left: the observed spark frequencies from 25 cells, plus a linear regression of the paired data. The slope was not significantly different than 1 (P = 0.49) and r2 = 0.32 (P = 0.0038). Right: average frequencies did not significantly vary (P = 0.38, paired t-test). C) Symmetrized average spark (n = 47 control and 67 8-CPT events), constructed by centering events at their peaks. D) The spatial and temporal profiles of average sparks showing in C.
(TIF)

Table S1 Observed spark parameters. Reported values are the average ± SEM of the numbers indicated in the table.
(TIF)

Table S2 Summary data for the balance of fluxes analysis for all treatments. (*statistically different from control, # from ISO, t-test, p<0.05).
(TIF)

## References

[1] Bers DM, Ziolo MT (2001) When is cAMP not cAMP? Effects of compartmentalization. CircRes 89: 373–375. 11532895

[2] Bers DM (2001) Excitation-Contraction Coupling and Cardiac Contractile Force: Kluwer Academic Publishers.

[3] Dibb KM, Graham HK, Venetucci LA, Eisner DA, Trafford AW (2007) Analysis of cellular calcium fluxes in cardiac muscle to understand calcium homeostasis in the heart. Cell Calcium 42: 503–512. 17509680 10.1016/j.ceca.2007.04.002

[4] Wang H, Kohr MJ, Traynham CJ, Wheeler DG, Janssen PM, et al (2008) Neuronal nitric oxide synthase signaling within cardiac myocytes targets phospholamban. Am J Physiol Cell Physiol 294: C1566–1575. 18400986 10.1152/ajpcell.00367.2007 PMC2771755

[5] Curran J, Brown KH, Santiago DJ, Pogwizd S, Bers DM, et al (2010) Spontaneous Ca waves in ventricular myocytes from failing hearts depend on Ca(2+)-calmodulin-dependent protein kinase II. J Mol Cell Cardiol 10.1016/j.yjmcc.2010.03.013 PMC2883657 20353795

[6] Grimm M, Brown JH (2010) Beta-adrenergic receptor signaling in the heart: role of CaMKII. J Mol Cell Cardiol 48: 322–330. 19883653 10.1016/j.yjmcc.2009.10.016 PMC2896283

[7] Curran J, Hinton MJ, Rios E, Bers DM, Shannon TR (2007) Beta-adrenergic enhancement of sarcoplasmic reticulum calcium leak in cardiac myocytes is mediated by calcium/calmodulin-dependent protein kinase. CircRes 100: 391–398. 10.1161/01.RES.0000258172.74570.e6 17234966

[8] Erickson JR, Joiner ML, Guan X, Kutschke W, Yang J, et al (2008) A dynamic pathway for calcium-independent activation of CaMKII by methionine oxidation. Cell 133: 462–474. 18455987 10.1016/j.cell.2008.02.048 PMC2435269

[9] Mangmool S, Shukla AK, Rockman HA (2010) {beta}-Arrestin-dependent activation of Ca2+/calmodulin kinase II after {beta}1-adrenergic receptor stimulation. J Cell Biol 10.1083/jcb.200911047 PMC2867304 20421423

[10] Oestreich EA, Wang H, Malik S, Kaproth-Joslin KA, Blaxall BC, et al (2007) Epac-mediated Activation of Phospholipase C{epsilon} Plays a Critical Role in beta-Adrenergic Receptor-dependent Enhancement of Ca2+ Mobilization in Cardiac Myocytes. JBiolChem 282: 5488–5495. 10.1074/jbc.M608495200 17178726

[11] Pereira L, Metrich M, Fernandez-Velasco M, Lucas A, Leroy J, et al (2007) The cAMP binding protein Epac modulates Ca2+ sparks by a Ca2+/calmodulin kinase signalling pathway in rat cardiac myocytes. J Physiol 583: 685–694. 17599964 10.1113/jphysiol.2007.133066 PMC2277038

[12] Erickson JR, Pereira L, Wang L, Han G, Ferguson A, et al (2013) Diabetic hyperglycaemia activates CaMKII and arrhythmias by O-linked glycosylation. Nature 10.1038/nature12537 PMC3801227 24077098

[13] Shannon TR, Ginsburg KS, Bers DM (2002) Quantitative assessment of the SR Ca2+ leak-load relationship. Circ Res 91: 594–600. 12364387 10.1161/01.res.0000036914.12686.28

[14] Bers DM, Bassani JW, Bassani RA (1993) Competition and redistribution among calcium transport systems in rabbit cardiac myocytes. CardiovascRes 27: 1772–1777. 10.1093/cvr/27.10.1772 8275522

[15] Bassani JW, Yuan W, Bers DM (1995) Fractional SR Ca release is regulated by trigger Ca and SR Ca content in cardiac myocytes. AmJPhysiol 268: C1313–C1319. 10.1152/ajpcell.1995.268.5.C1313 7762626

[16] Hobai IA, O'Rourke B (2001) Decreased sarcoplasmic reticulum calcium content is responsible for defective excitation-contraction coupling in canine heart failure. Circulation 103: 1577–1584. 11257088 10.1161/01.cir.103.11.1577

[17] Ziolo MT (2008) The fork in the nitric oxide road: cyclic GMP or nitrosylation? Nitric Oxide 18: 153–156. 18312860 10.1016/j.niox.2008.01.008 PMC2366797

[18] Wang H, Viatchenko-Karpinski S, Sun J, Gyorke I, Benkusky NA, et al (2010) Regulation of myocyte contraction via neuronal nitric oxide synthase: role of ryanodine receptor S-nitrosylation. J Physiol 588: 2905–2917. 20530114 10.1113/jphysiol.2010.192617 PMC2956906

[19] Kanai AJ, Mesaros S, Finkel MS, Oddis CV, Birder LA, et al (1997) Beta-adrenergic regulation of constitutive nitric oxide synthase in cardiac myocytes. Am J Physiol 273: C1371–1377. 9357783 10.1152/ajpcell.1997.273.4.C1371

[20] Salama G, Menshikova EV, Abramson JJ (2000) Molecular interaction between nitric oxide and ryanodine receptors of skeletal and cardiac sarcoplasmic reticulum. Antioxid Redox Signal 2: 5–16. 11232600 10.1089/ars.2000.2.1-5

[21] Sartoretto JL, Kalwa H, Pluth MD, Lippard SJ, Michel T (2011) Hydrogen peroxide differentially modulates cardiac myocyte nitric oxide synthesis. Proc Natl Acad Sci U S A 108: 15792–15797. 21896719 10.1073/pnas.1111331108 PMC3179126

[22] Kawasaki K, Smith RS Jr, Hsieh CM, Sun J, Chao J, et al (2003) Activation of the phosphatidylinositol 3-kinase/protein kinase Akt pathway mediates nitric oxide-induced endothelial cell migration and angiogenesis. Mol Cell Biol 23: 5726–5737. 12897144 10.1128/MCB.23.16.5726-5737.2003 PMC166338

[23] Haynes MP, Sinha D, Russell KS, Collinge M, Fulton D, et al (2000) Membrane estrogen receptor engagement activates endothelial nitric oxide synthase via the PI3-kinase-Akt pathway in human endothelial cells. Circ Res 87: 677–682. 11029403 10.1161/01.res.87.8.677

[24] Pereira L, Cheng H, Lao DH, Na L, van Oort RJ, et al (2013) Epac2 mediates cardiac beta1-adrenergic-dependent sarcoplasmic reticulum Ca2+ leak and arrhythmia. Circulation 127: 913–922. 23363625 10.1161/CIRCULATIONAHA.12.148619 PMC3690126

[25] Barouch LA, Harrison RW, Skaf MW, Rosas GO, Cappola TP, et al (2002) Nitric oxide regulates the heart by spatial confinement of nitric oxide synthase isoforms. Nature 416: 337–339. 11907582 10.1038/416337a

[26] Bovo E, Lipsius SL, Zima AV (2012) Reactive oxygen species contribute to the development of arrhythmogenic Ca(2)(+) waves during beta-adrenergic receptor stimulation in rabbit cardiomyocytes. J Physiol 590: 3291–3304. 22586224 10.1113/jphysiol.2012.230748 PMC3459043

[27] Ai X, Curran JW, Shannon TR, Bers DM, Pogwizd SM (2005) Ca2+/calmodulin-dependent protein kinase modulates cardiac ryanodine receptor phosphorylation and sarcoplasmic reticulum Ca2+ leak in heart failure. CircRes 97: 1314–1322. 10.1161/01.RES.0000194329.41863.89 16269653

[28] Ferrero P, Said M, Sanchez G, Vittone L, Valverde C, et al (2007) Ca2+/calmodulin kinase II increases ryanodine binding and Ca2+-induced sarcoplasmic reticulum Ca2+ release kinetics during beta-adrenergic stimulation. J Mol Cell Cardiol 43: 281–291. 17643448 10.1016/j.yjmcc.2007.05.022 PMC2045504

[29] Morimoto S, J OU, Kawai M, Hoshina T, Kusakari Y, et al (2009) Protein kinase A-dependent phosphorylation of ryanodine receptors increases Ca2+ leak in mouse heart. Biochem Biophys Res Commun 390: 87–92. 19781523 10.1016/j.bbrc.2009.09.071

[30] Gutierrez DA, Fernandez-Tenorio M, Ogrodnik J, Niggli E (2013) NO-dependent CaMKII activation during beta-adrenergic stimulation of cardiac muscle. Cardiovasc Res 100: 392–401. 23963842 10.1093/cvr/cvt201

[31] Hoch B, Meyer R, Hetzer R, Krause EG, Karczewski P (1999) Identification and expression of delta-isoforms of the multifunctional Ca2+/calmodulin-dependent protein kinase in failing and nonfailing human myocardium. Circ Res 84: 713–721. 10189359 10.1161/01.res.84.6.713

[32] Zhang R, Khoo MS, Wu Y, Yang Y, Grueter CE, et al (2005) Calmodulin kinase II inhibition protects against structural heart disease. NatMed 11: 409–417. 10.1038/nm1215 15793582

[33] Zhang T, Maier LS, Dalton ND, Miyamoto S, Ross J Jr, et al (2003) The deltaC isoform of CaMKII is activated in cardiac hypertrophy and induces dilated cardiomyopathy and heart failure. CircRes 92: 912–919. 10.1161/01.RES.0000069686.31472.C5 12676814

[34] Li JM, Gall NP, Grieve DJ, Chen M, Shah AM (2002) Activation of NADPH oxidase during progression of cardiac hypertrophy to failure. Hypertension 40: 477–484. 12364350 10.1161/01.hyp.0000032031.30374.32

[35] Bers DM (2002) Cardiac excitation-contraction coupling. Nature 415: 198–205. 11805843 10.1038/415198a

[36] Cutler MJ, Plummer BN, Wan X, Sun QA, Hess D, et al (2012) Aberrant S-nitrosylation mediates calcium-triggered ventricular arrhythmia in the intact heart. Proc Natl Acad Sci U S A 109: 18186–18191. 23071315 10.1073/pnas.1210565109 PMC3497770

[37] Traynham CJ, Roof SR, Wang H, Prosak RA, Tang L, et al (2012) Diesterified nitrone rescues nitroso-redox levels and increases myocyte contraction via increased SR Ca(2+) handling. PLoS One 7: e52005. 23300588 10.1371/journal.pone.0052005 PMC3531448

[38] Zhang X, Szeto C, Gao E, Tang M, Jin J, et al (2013) Cardiotoxic and cardioprotective features of chronic beta-adrenergic signaling. Circ Res 112: 498–509. 23104882 10.1161/CIRCRESAHA.112.273896 PMC3562387

