# Supplementary material for: Nitric Oxide-Dependent Activation of CaMKII Increases Diastolic Sarcoplasmic Reticulum Calcium Release in Cardiac Myocytes in Response to Adrenergic Stimulation
Source: PLoS One. 2014 Feb 3;9(2):e87495. doi: 10.1371/journal.pone.0087495 (PMC3911966; doi:10.1371/journal.pone.0087495)
Supplement: File S1 — File includes Figures S1–S5 and Tables S1–S2. (DOC) [file pone.0087495.s001.doc]

Supplementary Material

# Supplemental Material and Methods

## Ca Measurement

All experiments were performed at room temperature using our novel protocol shown in Figure 1S and as previously described . Before each leak protocol myocytes were stimulated electrically at 0.5 Hz for at least 20 pulses to assure that steady state calcium handling was achieved. The diastolic whole cell fluorescence (F_0_) between beats was collected. The diastolic [Ca]_i_  ([Ca]_d_) under each relevant condition was determined in separate experiments using calibrated fura-2 fluorescence (data not shown). This [Ca]_d_ did not statistically vary between treatments, and was generally found to be approximately 120 nM. The fluo-4 fluorescence (F) during the subsequent protocol was calibrated by using a pseudoratio:


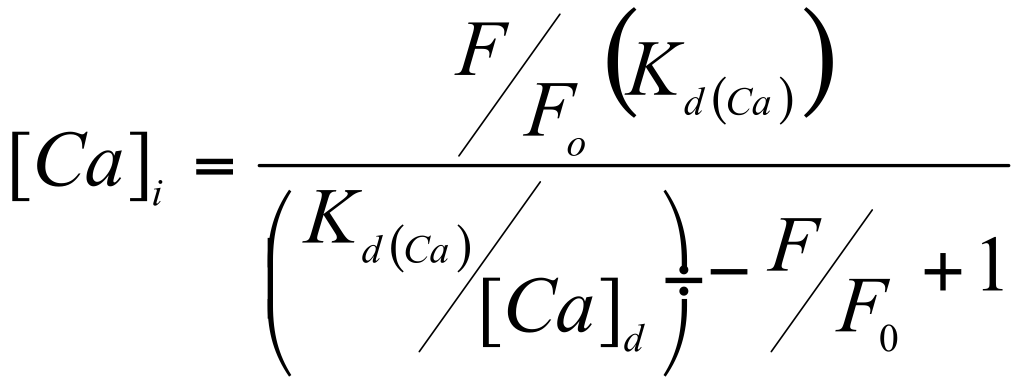


where K_d(Ca)_ of fluo-4 was 1.1 µM.

SR Ca Leak Measurement

SR Ca^2+^ leak was measured as described in the main manuscript.

Spontaneous Ca Wave Measurement

Spontaneous Ca^2+^ waves were measured as described in the main manuscript.

## Spark measurements in intact myocytes

Flou-4 loaded rabbit myocytes were field-stimulated at 1 Hz for 2 minutes on a Biorad MRC 1000 confocal miscroscope. The stimulator was then turned off and 1 linescan recorded every 4 seconds for 32 seconds (i.e. a total of 8 images during the post-rest). The temporal resolution within each image was 2 ms per line. Each cell was tested twice, first in the absence and then in the presence of 2 μM 8-CPT, without a change in microscope settings. Prior to each protocol, 25 beats were given at 0.5 Hz in 8-CPT-free solution, to ensure that the selected cell had similar steady-state [Ca^2+^] transients.

For the analysis of paired data, a two-tailed t-test was used. For unpaired data, a two-tailed t-test with Welch correction (for unequal variances) was used. Signal mass was calculated at the time of peak, using a formula from Chandler et al In Table S1, the lowest n values represent data where the monoexponential or Gaussian fits could be successfully performed. Differences were considered statistically significant when P < 0.05.

## Balance of Fluxes Analysis

Transport fluxes were analyzed by the method Bassani et al **.** Rabbit cells were field stimulated at least 20 times to obtain steady-state [Ca]_i_ transients in the Ca-dependent fluorescent dye fluo-4. This was followed by application of 10 mM caffeine to cause release of the Ca within the SR into the cytosol. Cells were exposed to caffeine in 0 Na-0 Ca normal Tyrode solution (0-0 NT) for 2 seconds followed by caffeine in nominally Ca-free normal Tyrode with Na present (0 Ca NT).

Free cytosolic [Ca] was converted to [Ca]_SRT_ using well established cytosolic buffering parameters . The derivative of the decline in [Ca]_SRT_ over time was determined at each time point after fitting the data to a declining exponential.

The slow decline of [Ca]_SRT_ in caffeine in 0-0 Ca NT was considered to be a combination of SL Ca pump-mediated Ca extrusion and mitochondrial Ca uptake. The rate of decline in caffeine in 0 Ca NT was this flux plus the extrusion of Ca via obligatory Na-Ca exchange (NCX). NCX activity was, therefore, calculated as the rate of decline in caffeine in 0 Ca NT with Ca minus the rate of decline in caffeine in 0-0 NT.

Finally the rates of decline, calculated as above, were taken as a function of their respective cytosolic free Ca values and fit to the standard Michaelis-Menton equation:

V=V_max_/(1+(K_m_/[Ca]_i_)^H^

where V is the rate of transport, Vmax is the maximum rate of transport, Km is [Ca]i at the half maximum rate, and H is the Hill coefficient for the NCX and the SR Ca pump, respectively.

The fitted parameters are used to calculate the integrated transport rate and the integrated transport during a average decline under the indicated conditions.

Statistical Analysis

Data are reported as mean ± SEM. Student t test was applied when appropriate. P<0.05 was considered statistically significant. To compare DAF-2 dependent fluorescence a non-parametric Spearman correlation test was conducted. The Spearman r-values are reported as an index of correlation of NO production with time.

# RESULTS

## Balance of Fluxes Analysis Reveals a Role for NOS1 Signaling During Inotropy

It is important to investigate whether NO may have other effects on EC coupling beyond modulating RyR behavior. For example, Kohr et al have found that ONOO^-^ signaling acts to increase SR Ca uptake by promoting phospholamban dissociation from SERCA, and this signaling requires NOS1 . Therefore we applied the balance of Ca fluxes analysis described by Bassani et al to uncover how NO and inhibition of its formation altered the pertinent Ca fluxes.

Figure S2 shows that at the same [Ca]_SRT_ (171 µM, Figure S2a) regardless of treatment we observed the expected increase in the gain of EC coupling, defined here at the integral of SR Ca flux (i.e. Ca released from the SR at steady-state) divided by the integral of NCX and SL mediated Ca fluxes (i.e. total Ca influx at steady-state), when ISO was present (1.1 ± 0.17, 3.7 ± 0.6, 4.4 ± 0.6, 3.6 ± 0.5, 4.2 ± 0.5). This is emphasized in the theoretical curves fit to our experimental data (see Table S2) for both SERCA- and NCX-mediate uptake kinetics (Figures S2c and S2d). Interestingly, when in the presence of SNAP alone the SERCA-mediated uptake was slightly suppressed below that of control. This likely reflects the increased SR Ca leak observed by SNAP stimulation (Figure 5, main text). As the SR Ca leak increases the SERCA-mediated influx is opposed by this larger efflux out of the SR. This would have the effect of lowering its effective V_max_ of uptake. Figure S2f shows the expected result of diminished NCX transport as myocyte contraction becomes more and more dependent upon SR Ca-mediated fluxes during increased inotropy.

Interestingly, when we inhibited NO formation the SERCA-mediated uptake was blunted in the presence of ISO. This was particularly evident when we examined the relative velocity of uptake at 250 nM [Ca]_i_, where NOS1 inhibition by SMLT significantly slowed velocity when compared with ISO (98 ± 5.8 µM s^-1^ and 133 ± 19, relatively, Figure S3E). Inhibition by L-NAME trended downward but did not attain statistical significance (110 ± 12 µM s^-1^, p = 0.08), while inhibition of NOS3 by L-NIO had no effect (125 ± 10 µM s^-1^). This suggests that NOS1 is somehow mediating at least a portion of the increased SERCA uptake kinetics observed with β-AR stimulation. This data is in line with Kohr et al who found a similar NOS1-dependent effect SR Ca uptake . The data further suggest that the inhibition by NO maybe a local response as generation of NO by SNAP addition had no effect upon uptake (Figure S2e).

## CaMKII-dependent Leak Is Not Mediated Through NADPH Oxidase

In order to further test whether known, alternative second messenger pathways play a role in the observed response to ISO we set out to determine if ROS plays a role in the acute CaMKII-dependent leak. We stimulated myocytes with ISO in the presence of the NADPH oxidase inhibitor, DPI (250 nM). Figure S3 shows that DPI is unable to shift the leak/load away from that observed in ISO. When selecting myocytes for similar [Ca]_SRT_ (112 µM, Figure S3B) the leak at that load was significantly higher in ISO and ISO plus DPI when compared with control (12.2 ± 0.9, 11.8 ± 2.6, 4.1 ± 0.9 µM, respectively). Similarly, when matching myocytes by similar leak (7.5 µM, Figure S3C) the [Ca]_SRT_ needed to induce that leak was significantly lower in ISO and ISO plus DPI versus control (104 ± 9, 115 ± 14, 178 ± 6 µM, respectively). From this data we conclude that NADPH-generated ROS does not mediate the acute of effects of CaMKII on SR Ca leak.

## CaMKII-dependent Leak Is Not Mediated Through EPAC

In order to test for the possible mediation of the increase in SR Ca leak by EPAC, we evaluated the leak-load relationship in the presence of 2 μM of the EPAC activator 8-CPT. 8-CPT had no effect upon the relationship (Figure S4A). When data were matched such that [Ca]_SRT_ was the same for all treatments, the resultant leaks did not increase (Figure S4B) nor did the load needed to generate the same leak change when data were matched in the opposite manner (Figure S4C).

Since, in contrast to previous measurements in small rodents , EPAC activation in rabbits did not affect the SR Ca leak. We further tested for the effects of EPAC activation by measuring Ca^2+^ sparks. Cells were pre-paced at 1 Hz for two minutes, thus achieving a reasonable SR Ca load, and then electrical stimulation was turned off and the measurements performed for 32 seconds. For any given cell, this was done first in the absence and then in the presence of 8-CPT, so that results would be paired.

Figure S5A are representative linescan images from two different sparking cells. Control denotes the initial measurements (i.e. before 8-CPT application) whereas 8-CPT indicates the same protocol repeated after 2 minutes of uninterrupted EPAC activation. White arrows indicate detected sparks. Post-rest times are indicated in white. Figure S2b summarizes the spark frequency observations for 25 cells. (See Table S1 for full summary of the spark morphology data.) In all cases, no significant differences were observed between the average parameters in the control protocol vs. the 8-CPT protocol. Likewise, the slope of the best linear regression for the spark frequency was not significantly different from 1 (P=0.49) and the correlation was evident (Figure S5B, Pearson r^2^ = 0.32; P=0.0038). For example, the same myocytes which under control conditions had a spark frequency of 0.77 ± 0.04 sparks 100 µm^-1^ s^-1^ had a spark frequency of 0.75 ± 0.22 parks 100 µm^-1^ s^-1^ after being treated with 8-CPT (figure S2b).

Finally, we constructed average sparks by centering the extractable events at their peaks and symmetrizing them (see Figure S5C, from 47 control and 67 8-CPT events). The temporal and spatial profiles of the averaged sparks are compared in Figure S2d. Once again, no differences were observed between the two conditions.

Angiotensin has No Effect Upon SR Ca Leak Rate

It has been suggested that β-arrestin may be involved in the activation of CaMKII as part of the adrenergic pathway . As a preliminary investigation into the possible role of this molecule in the pathway described in the current manuscript, we performed our SR Ca leak protocol in the presence of angiotensin II (ATII), a strong activator of β-arrestin. ATII had no effect upon the SR Ca leak-load relationship. There was no significant increase in SR Ca leak when measure in cells matched for [Ca]_SRT_ nor was there an increase in the SR [Ca] needed to generate the same leak. The results indicate that if β-arrestin is involved in the increased SR Ca leak measured as part of adrenergic response described here and in the main text, it is likely specific to the β_1_-AR receptor which mediates this effect

.

REFERENCES

1. Curran, J., et al., *Beta-adrenergic enhancement of sarcoplasmic reticulum calcium leak in cardiac myocytes is mediated by calcium/calmodulin-dependent protein kinase.* Circ.Res., 2007. **100**(3): p. 391-398.

2. Shannon, T.R., K.S. Ginsburg, and D.M. Bers, *Quantitative assessment of the SR Ca2+ leak-load relationship.* Circ Res, 2002. **91**(7): p. 594-600.

3. Chandler, W.K., S. Hollingworth, and S.M. Baylor, *Simulation of calcium sparks in cut skeletal muscle fibers of the frog.* J Gen Physiol, 2003. **121**(4): p. 311-24.

4. Bassani, R.A., J.W. Bassani, and D.M. Bers, *Relaxation in ferret ventricular myocytes: unusual interplay among calcium transport systems.* J.Physiol, 1994. **476**(2): p. 295-308.

5. Hove-Madsen, L. and D.M. Bers, *Passive Ca buffering and SR Ca uptake in permeabilized rabbit ventricular myocytes.* Am J Physiol, 1993. **264**(3 Pt 1): p. C677-86.

6. Wang, H., et al., *Neuronal nitric oxide synthase signaling within cardiac myocytes targets phospholamban.* Am J Physiol Cell Physiol, 2008. **294**(6): p. C1566-75.

7. Kohr, M.J., et al., *Nitroxyl enhances myocyte Ca2+ transients by exclusively targeting SR Ca2+-cycling.* Front Biosci (Elite Ed), 2010. **2**: p. 614-26.

8. Bassani, J.W., R.A. Bassani, and D.M. Bers, *Relaxation in rabbit and rat cardiac cells: species-dependent differences in cellular mechanisms.* J.Physiol, 1994. **476**(2): p. 279-293.

9. Oestreich, E.A., et al., *Epac-mediated Activation of Phospholipase C{epsilon} Plays a Critical Role in beta-Adrenergic Receptor-dependent Enhancement of Ca2+ Mobilization in Cardiac Myocytes.* J.Biol.Chem., 2007. **282**(8): p. 5488-5495.

10. Mangmool, S., A.K. Shukla, and H.A. Rockman, *{beta}-Arrestin-dependent activation of Ca2+/calmodulin kinase II after {beta}1-adrenergic receptor stimulation.* J Cell Biol, 2010.
