# Supplementary figures and images for: Nitric Oxide-Dependent Activation of CaMKII Increases Diastolic Sarcoplasmic Reticulum Calcium Release in Cardiac Myocytes in Response to Adrenergic Stimulation
Source: PLoS One. 2014 Feb 3;9(2):e87495. doi: 10.1371/journal.pone.0087495 (PMC3911966; doi:10.1371/journal.pone.0087495)

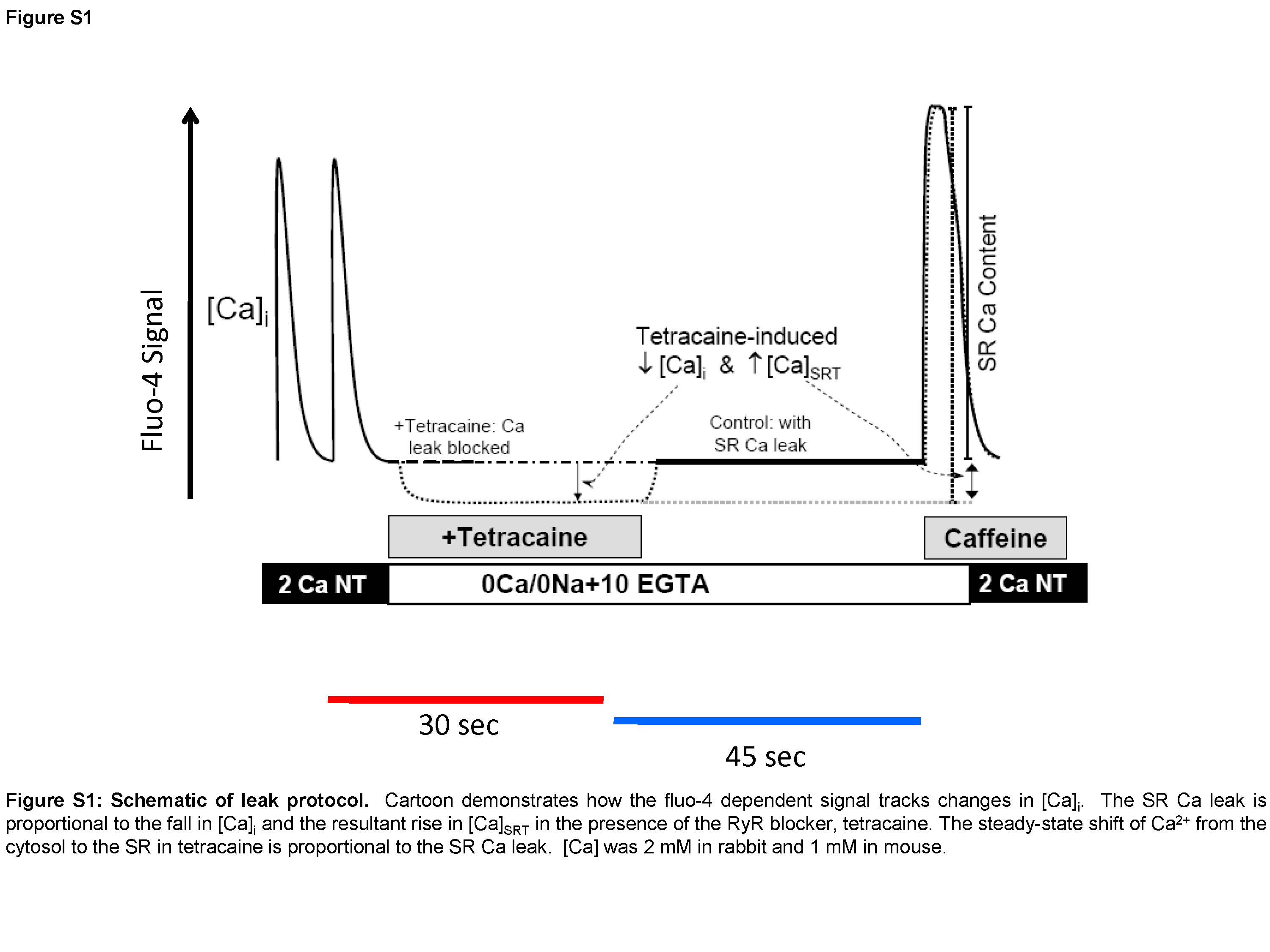

Supplement: Figure S1 — Schematic of leak protocol. Cartoon demonstrates how the fluo-4 dependent signal tracks changes in [Ca]i. The SR Ca leak is proportional to the fall in [Ca]i and the resultant rise in [Ca]SRT in the presence of the RyR blocker, tetracaine. The steady-state shift of Ca2+ from the cytosol to the SR in tetracaine is proportional to the SR Ca leak. [Ca] was 2 mM in rabbit and 1 mM in mouse. (TIF) [file pone.0087495.s002.tif]

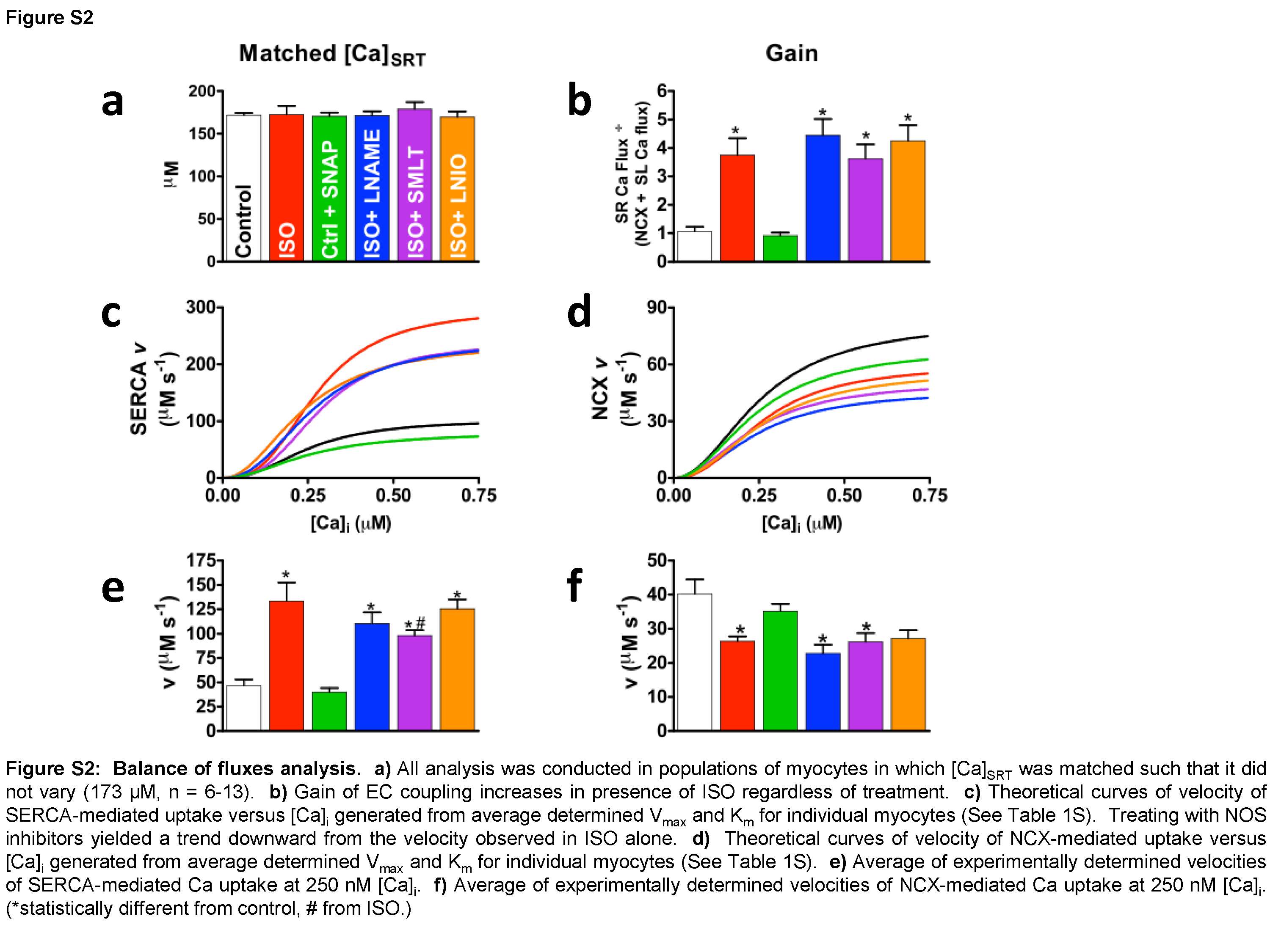

Supplement: Figure S2 — Balance of fluxes analysis. a) All analysis was conducted in populations of myocytes in which [Ca]SRT was matched such that it did not vary (173 µM, n = 6–13). b) Gain of EC coupling increases in presence of ISO regardless of treatment. c) Theoretical curves of velocity of SERCA-mediated uptake versus [Ca]i generated from average determined Vmax and Km for individual myocytes (See Table 1S). Treating with NOS inhibitors yielded a trend downward from the velocity observed in ISO alone. d) Theoretical curves of velocity of NCX-mediated uptake versus [Ca]i generated from average determined Vmax and Km for individual myocytes (See Table 1S). e) Average of experimentally determined velocities of SERCA-mediated Ca uptake at 250 nM [Ca]i. f) Average of experimentally determined velocities of NCX-mediated Ca uptake at 250 nM [Ca]i. (*statistically different from control, # from ISO.) (TIF) [file pone.0087495.s003.tif]

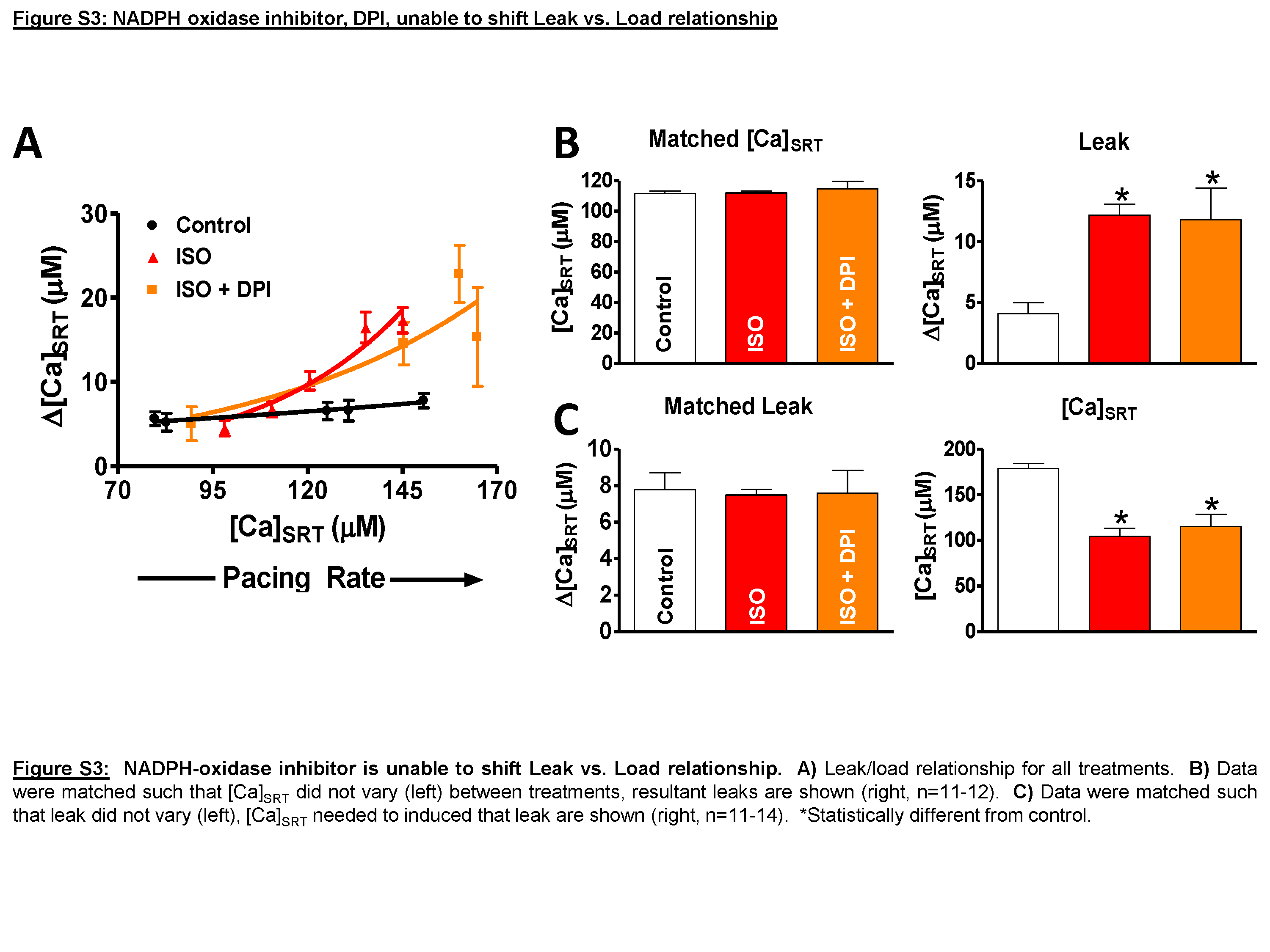

Supplement: Figure S3 — NADPH-Oxidase inhibitor is unable to shift leak vs. load relationship. A) Leak/load relationship for all treatments. B) Data were matched such that [Ca]SRT did not vary (left) between treatments, resultant leaks are show (right, n = 11–12). C) Data were matched such that leak did vary (left), [Ca]SRT needed to induce that leak are shown (right, n = 11–14). *Statistically different from control. (TIF) [file pone.0087495.s004.tif]

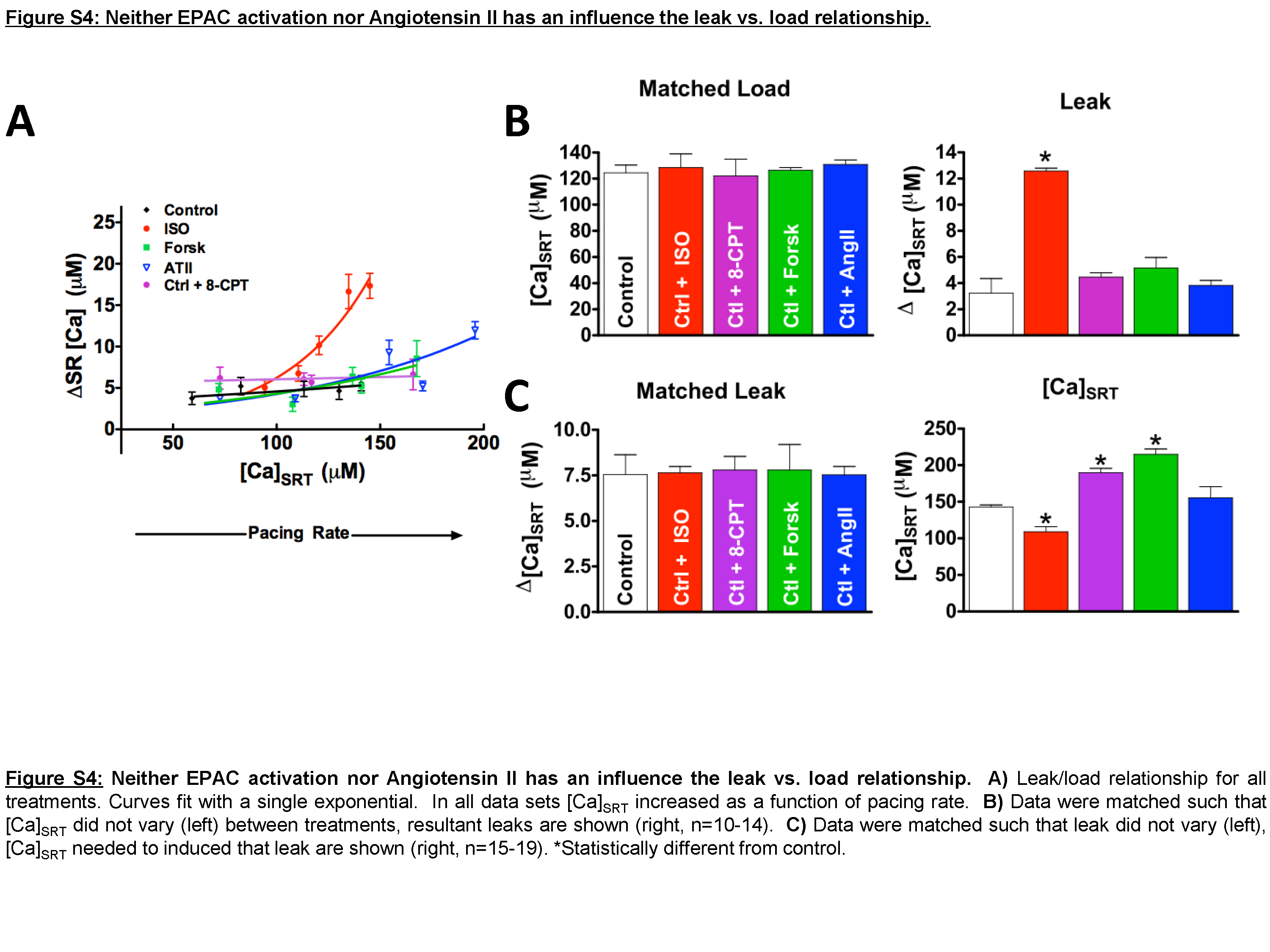

Supplement: Figure S4 — Neither EPAC activation nor Angiotensin II has an influence the leak vs. load relationship. A) Leak/load relationship for all treatments. Curves fit with a single exponential. In all data sets [Ca]SRT increased as a function of pacing rate. B) Data were matched such that [Ca]SRT did not vary (left) between treatments, resultant leaks are shown (right, n = 10–14). C) Data were matched such that leak did not vary (left), [Ca]SRT needed to induced that leak are shown (right, n = 15–19). *Statistically different from control. (TIF) [file pone.0087495.s005.tif]

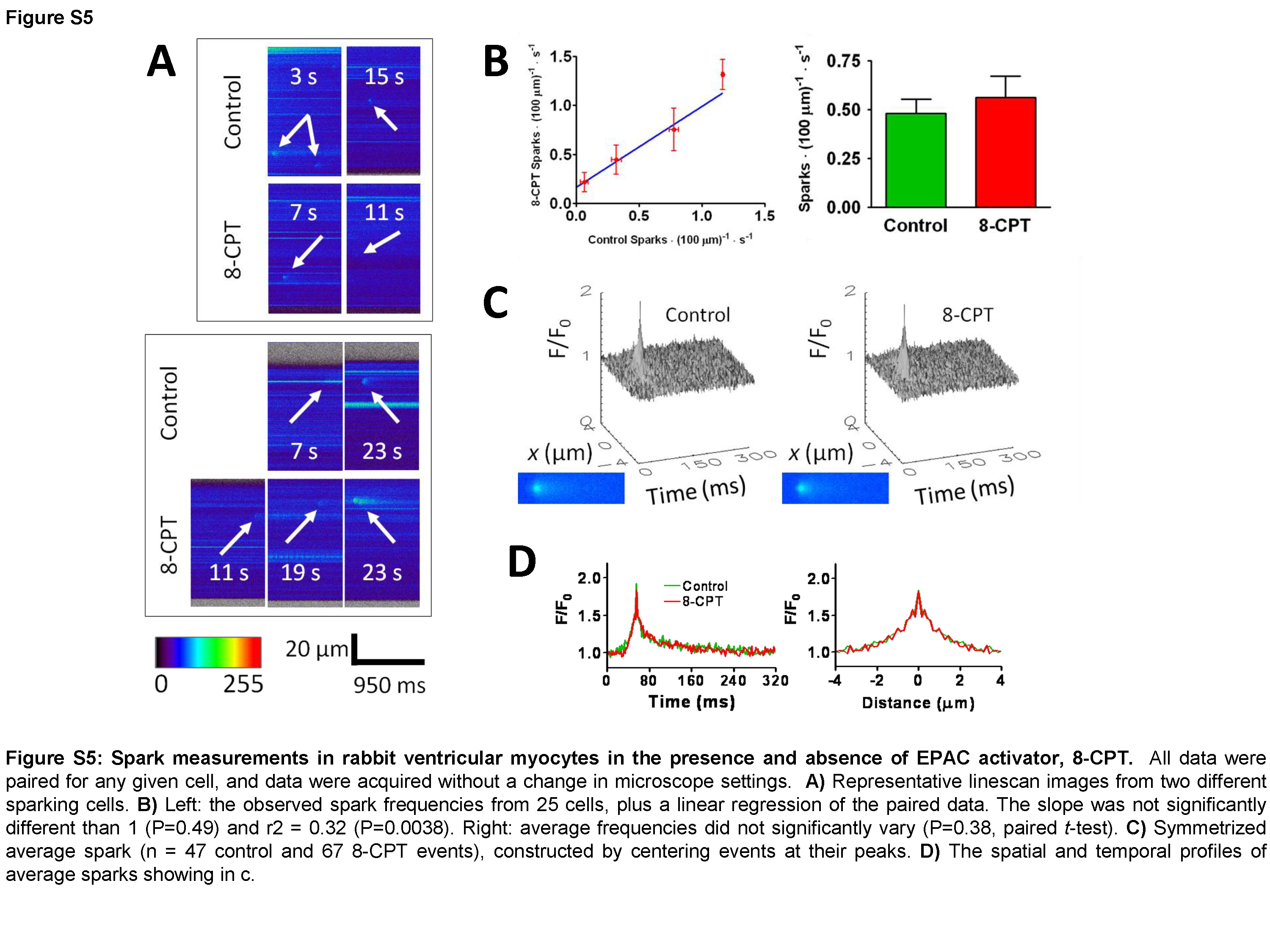

Supplement: Figure S5 — Spark measurements in rabbit ventricular myocytes in the presence and absence of EPAC activator, 8-CPT. All data were paired for any given cell, and data were acquired without a change in microscope settings. A) Representative linescan images from two different sparking cells. B) Left: the observed spark frequencies from 25 cells, plus a linear regression of the paired data. The slope was not significantly different than 1 (P = 0.49) and r2 = 0.32 (P = 0.0038). Right: average frequencies did not significantly vary (P = 0.38, paired t-test). C) Symmetrized average spark (n = 47 control and 67 8-CPT events), constructed by centering events at their peaks. D) The spatial and temporal profiles of average sparks showing in C. (TIF) [file pone.0087495.s006.tif]
